# Bacterial diversity of bacteriomes and organs of reproductive, digestive and excretory systems in two cicada species (Hemiptera: Cicadidae)

**DOI:** 10.1371/journal.pone.0175903

**Published:** 2017-04-24

**Authors:** Zhou Zheng, Dandan Wang, Hong He, Cong Wei

**Affiliations:** 1 Key Laboratory of Plant Protection Resources and Pest Management, Ministry of Education, College of Plant Protection, Northwest A&F University, Yangling, Shaanxi, China; 2 College of Forestry, Northwest A&F University, Yangling, Shaanxi, China; University of Minnesota, UNITED STATES

## Abstract

Cicadas form intimate symbioses with bacteria to obtain nutrients that are scarce in the xylem fluid they feed on. The obligate symbionts in cicadas are purportedly confined to specialized bacteriomes, but knowledge of bacterial communities associated with cicadas is limited. Bacterial communities in the bacteriomes and organs of reproductive, digestive and excretory systems of two cicada species (*Platypleura kaempferi* and *Meimuna mongolica*) were investigated using different methods, and the bacterial diversity and distribution patterns of dominant bacteria in different tissues were compared. Within each species, the bacterial communities of testes are significantly different from those of bacteriomes and ovaries. The dominant endosymbiont *Candidatus* Sulcia muelleri is found not only in the bacteriomes and reproductive organs, but also in the “filter chamber + conical segment” of both species. The transmission mode of this endosymbiont in the alimentary canal and its effect on physiological processes merits further study. A novel bacterium of Rhizobiales, showing ~80% similarity to *Candidatus* Hodgkinia cicadicola, is dominant in the bacteriomes and ovaries of *P*. *kaempferi*. Given that the genome of *H*. *cicadicola* exhibits rapid sequence evolution, it is possible that this novel bacterium is a related endosymbiont with beneficial trophic functions similar to that of *H*. *cicadicola* in some other cicadas. Failure to detect *H*. *cicadicola* in *M*. *mongolica* suggests that it has been subsequently replaced by another bacterium, a yeast or gut microbiota which compensates for the loss of *H*. *cicadicola*. The distribution of this novel Rhizobiales species in other cicadas and its identification require further investigation to help establish the definition of the bacterial genus *Candidatus* Hodgkinia and to provide more information on sequence divergence of related endosymbionts of cicadas. Our results highlight the complex bacterial communities of cicadas, and are informative for further studies of the interactions and co-evolution of insect-microbial symbioses in Cicadoidea.

## Introduction

Phytophagous sap sucking insects in the insect order Hemiptera usually feed on nutritionally deficient xylem or phloem diets [1]. How do these insects survive with such a nutritionally poor diet? The answer possibly lies in the microbial symbionts with which they coexist [2,3].

In hemipterans, such symbionts include primary (obligate) and secondary (facultative) groups. The most distinctive group is primary symbionts, such as *Candidatus* Buchnera aphidicola in aphids [4] and *Candidatus* Sulcia muelleri (hereafter *Sulcia muelleri*) in Auchenorrhyncha [5]. They are usually confined in the bacteriomes, specialized clusters of cells that house endosymbionts and protect them against the host immune system [6,7]. In turn, these primary symbionts provide insects with essential nutrients that are not sufficient in their natural diet and cannot be synthesized by the insect hosts [8–10]. As a consequence of vertical transmission, primary symbionts co-evolve with their insect hosts [6,11–15].

In addition to these primary symbionts, various secondary symbionts such as *Rickettsia*, *Wolbachia* and *Cardinium*, have been sporadically recorded in Hemiptera. Secondary symbionts are more recent in origin, and they can be found in the hemolymph, salivary glands [16–18], Malpighian tubules [19], reproductive organs [17,18], bacteriomes [20], and fat body cells of insects [14]. They can be transmitted both vertically and horizontally [21]. Secondary symbionts have been reported to take part in reproductive manipulations, which may improve their own transmission and help the host to increase fitness under specific environmental conditions, such as responding to heat stress or chemical insecticides [22–26].

The superfamily Cicadoidea is one of the lineages of Hemiptera, which are well known for the loud calling songs generated by the male adults [27]. Both nymphal and adult cicadas feed on xylem sap, which is an extremely diluted food source limited in carbohydrates, amino acids, and vitamins [28]. As a result, they confront serious nutritional deficiencies [6,29,30]. Missing nutrients supplied by bacteria are mainly derived from the primary ensymbionts *S*. *muelleri*, or on *S*. *muelleri* together with *Candidatus* Hodgkinia cicadicola (hereafter *Hodgkinia cicadicola*), located in the bacteriomes of some species [2,30]. Coexistence of these endosymbionts with cicadas is a complementary consequence in their biosynthetic capabilities [2]. Previous studies on bacteria associated with cicadas have focused only on a few cicada species and relied on genomic sequencing [2,30], high-throughput proteomics [30], Fluorescence In Situ Hybridization (FISH) [6], Denaturing Gradient Gel Electrophoresis (DGGE) [31] and Restriction Fragment Length Polymorphism (RFLP) [32]. To date, information on the bacterial communities of the bacteriomes and reproductive organs of cicadas is extremely limited and merits more investigation.

In the current study, we initially investigate the bacterial communities residing in the bacteriomes of females of two cicada species, *Platypleura kaempferi* (Fabricius) and *Meimuna mongolica* (Distant), using 16S rRNA Restriction Fragment Length Polymorphism (RFLP) analysis. Then we further confirm the distributions of the dominant bacteria in other tissues of the host, i.e., the bacteriomes of males, salivary glands, alimentary canal, Malpighian tubules, ovaries, and testes, using diagnostic PCR. Furthermore, we analyze the bacterial communities residing in the bacteriomes of both sexes and reproductive organs of these two cicada species using Illumina high-throughput sequencing technology. We aim to address the following questions: 1) What are the composition and diversity of bacterial communities in the bacteriomes and reproductive organs of these two cicada species? 2) Does *H*. *cicadicola* co-exist with *S*. *muelleri* in these two cicada species? 3) Do cicada species, sexes and tissues have any influence on the bacterial community composition and diversity? 4) What differences can be revealed between the bacterial communities investigated by using different research methods?

## Results

### Bacterial composition of bacteriomes of female cicadas analyzed by RFLP

In the bacteriome-clone libraries of *P*. *kaempferi* and *M*. *mongolica*, 200 and 198 positive clones were selected, and digested with *Afa* I and *Hha* I restriction endonucleases, respectively. We obtained 67 and 58 main RFLP profiles for each clone library, and in total 113 and 66 representative clones were sequenced, respectively. Their blast results are summarized in Tables 1 and 2.

**Table 1:** NCBI BLAST results for the 16S rRNA-RFLP sequences of the representative clones isolated from the bacteriomes of female *P*. *kaempferi*.

No. of representative clones	GenBank accession No.	Clone numbers (the % in clone library)	Closest match species in GenBank	Identity to closest match (%)
**Clone PK-41**	KR911839	91 (45.50%)	*Rickettsia* symbiont of *Nephotettix cincticeps* (AB702995.1) (Proteobacteria)	99%
**Clone PK-138**	KR911841	60 (30.00%)	*Hodgkinia cicadicola* (NR_074753.1) (Proteobacteria)	81%
**Clone PK-121**	KR911840	34 (17.00%)	*Hodgkinia cicadicola* (NR_074753.1) (Proteobacteria)	79%
**Clone PK-132**	KR911843	10 (5.00%)	*Hodgkinia cicadicola* (NR_074753.1) (Proteobacteria)	80%
**Clone PK-14**	KR911842	2 (1.00%)	*Hodgkinia cicadicola* (NR_074753.1) (Proteobacteria)	83%
**Clone PK-166**	KR911844	3 (1.50%)	*Meiothermus cerbereus* (NR_026421.1) (Deinococcus-Thermus)	99%

**Table 2:** NCBI BLAST results for the 16S rRNA-RFLP sequences of the representative clones isolated from the bacteriomes of female *M*. *mongolica*.

No. of representative clones	GenBank accession No.	Clone numbers (the % in clone library)	Closest match species in GenBank	Identity to closest match (%)
**Clone MM-17**	KR911848	103 (52.02%)	*Sulcia muelleri* (EU930843.1) (Bacteroidetes)	99%
**Clone MM-2**	KR911845	57 (28.79%)	*Spiroplasma* sp. (DQ452375.1) (Tenericutes)	96%
**Clone MM-127**	KR911846	17 (8.59%)	*Spiroplasma* sp. (DQ452375.1) (Tenericutes)	95%
**Clone MM-44**	KR911849	14 (7.07%)	*Meiothermus cerbereus* (NR_026421.1) (Deinococcus-Thermus)	99%
**Clone MM-84**	KR911850	6 (3.03%)	*Rhodococcus* sp. (KF150201.1) (Actinobacteria)	99%
**Clone MM-3**	KR911847	1 (0.51%)	*Bacillus* sp. (FJ764775.1) (Firmicutes)	97%

**Fig 1:**
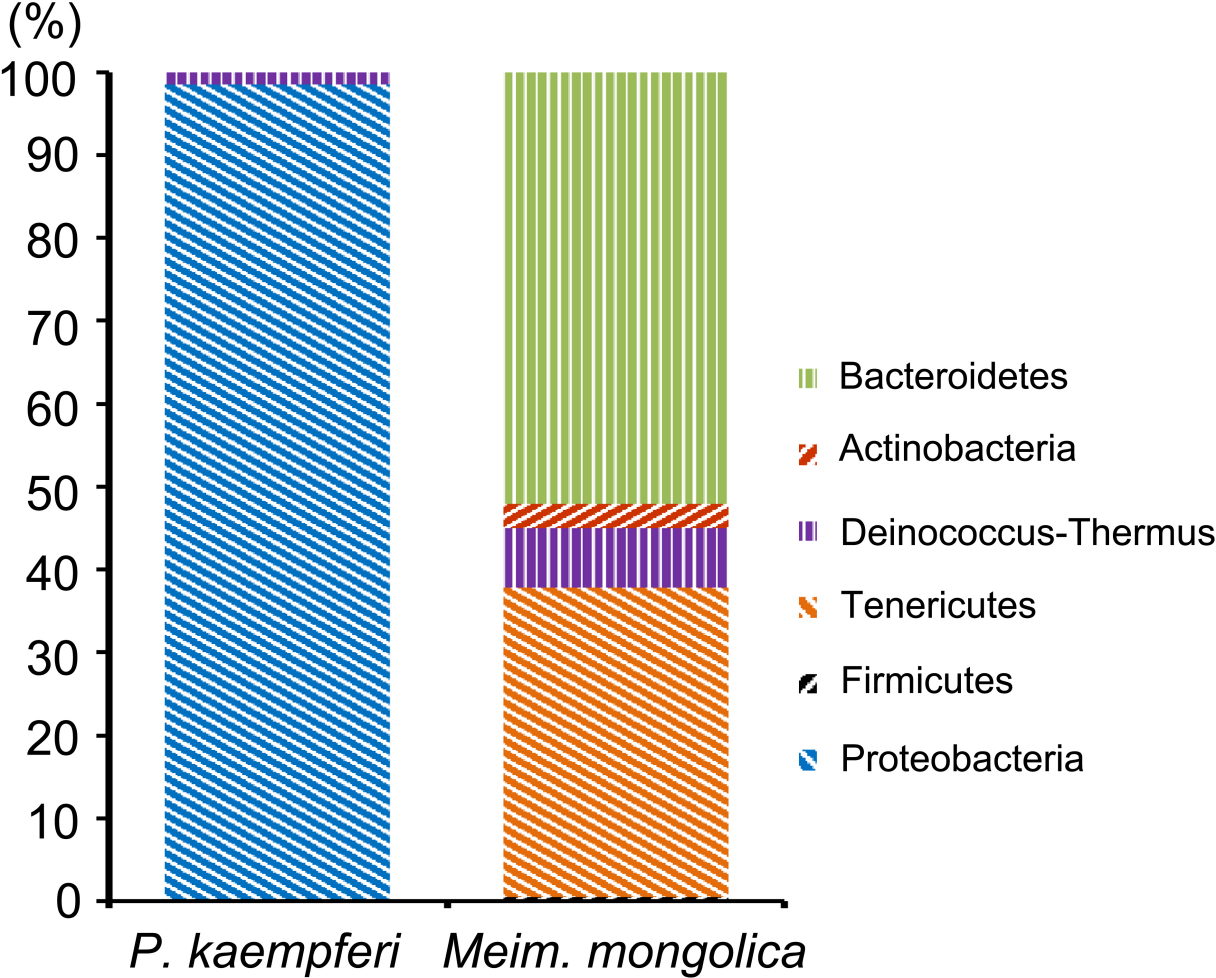
Bacterial composition of the bacteriomes-clone libraries of female *P*. *kaempferi* and *M*. *mongolica* at the phylum level.

Bacteria in the bacteriomes of female *P*. *kaempferi* belong to two major phyla (Table 1 and Fig 1), i.e., Proteobacteria (98.50%) and Deinococcus-Thermus (1.50%). At the species level, a novel Rhizobiales bacterium (KR911840-KR911843) (53.00%) which shows ~80% similarity to *H*. *cicadicola* is the most dominant species. *Rickettsia* sp. (KR911839) (45.50%), previously detected from the green rice leafhopper *Nephotettix cincticeps* (Uhler) (AB702995.1), is the second dominant bacterium. *Meiothermus cerbereus* (KR911844) (1.50%) is the single bacterial species belonging to the Deinococcus-Thermus.

Bacterial communities residing in the bacteriomes of female *M*. *mongolica* are classified into five species. *Sulcia muelleri* (KR911848) (52.02%) is the most abundant species followed by *Spiroplasma* sp. (KR911845 and KR911846) (37.38%). The abundance of *Meiothermus cerbereus* (KR911849) (7.07%) is relatively low. The remaining two bacteria species, *Rhodococcus* sp. (KR911850) (3.03%) and *Bacillus* sp. (KR911847) (0.51%), are both in very low concentrations (Table 2 and Fig 1).

### Bacterial diversity and phylogeny associated with bacteriomes of females analyzed by RFLP

**Fig 2:**
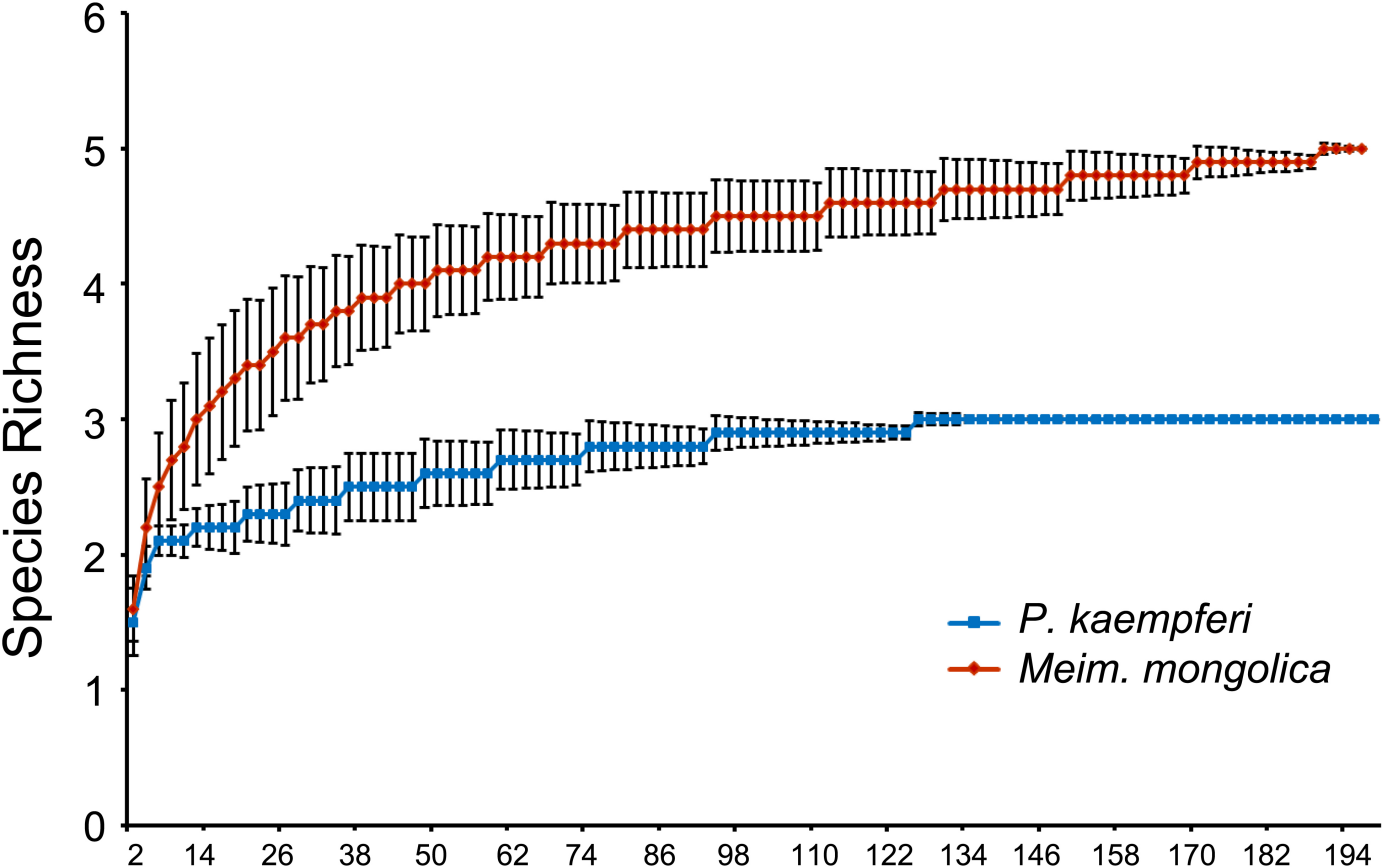
Rarefaction analyses of 16S rRNA gene libraries constructed from the bacteriomes of female *P*. *kaempferi* and *M*. *mongolica*.

The rarefaction curves for both clone libraries of *P*. *kaempferi* and *M*. *mongolica* reach plateaus at a 3% difference between sequences (95% confidence) (Fig 2). This suggests that the number of clones sampled is sufficient to provide an accurate estimation of bacterial diversity in the bacteriomes of female cicadas. Among the diversity indices (Table 3), Coverage C of the two clone libraries reaches 1.000 and 0.995, respectively; the species richness and Shannon indices of *M*. *mongolica* are higher than those of *P*. *kaempferi*, but the Simpson index is the opposite.

**Table 3:** Diversity indices of the 16S rRNA gene clone libraries constructed from the bacteriomes of female *P*. *kaempferi* and *M*. *mongolica* (95% confidence interval).

Cicada species	Number of clones (*N*)	Bacterial species	Shannon index	Simpson index	Coverage C
***P*. *kaempferi***	200	3	0.758	0.486	1.000
***M*. *mongolica***	198	5	1.028	0.413	0.995

**Fig 3:**
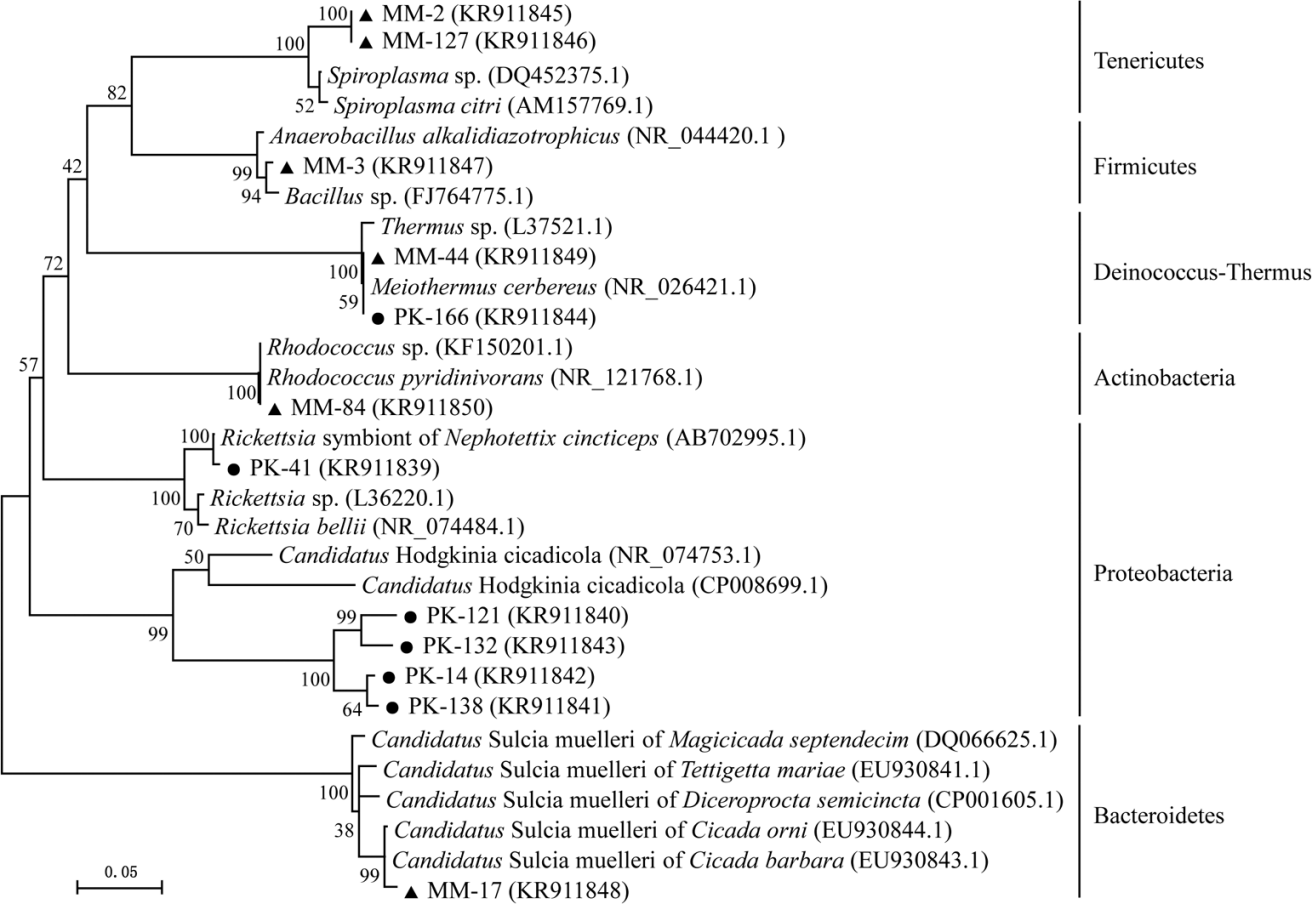
The ML phylogenetic tree based on bacterial 16S rRNA gene sequences obtained from the bacteriomes of female *P*. *kaempferi* and *M*. *mongolica*, including selected database sequences. This tree was generated using the Maximum Likelihood with 2,000 bootstrap replicates and Kimura 2-parameter model in MEGA5.0 software. The representative clones identified are listed in boldface type followed by GenBank accession numbers. Bacterial phyla are indicated on the right side. The scale bar represents 0.05 substitutions per nucleotide site. The representative clone sequences from the bacteriomes of female *P*. *kaempferi* are presented with dark spots, and the representative clone sequences from the bacteriomes of female *M*. *mongolica* are presented with dark triangles.

A Maximum Likelihood tree constructed using sequences of the 12 representative clones from the two clone libraries and their best matched sequences from GenBank revealed that the detected bacteria are affiliated with six phyla (Fig 3). An additional Maximum Likelihood tree constructed using sequences of the four clones of the novel Rhizobiales bacterium obtained in this study and other known sequences of Rhizobiales from GenBank, confirmed that this novel Rhizobiales bacterium is closely related with *H*. *cicadicola* (S1 Fig).

### Localization of dominant bacteria in different tissues detected by diagnostic PCR

Our diagnostic PCR performed on different tissues of *P*. *kaempferi* shows that: 1) both *S*. *muelleri* (KR911848) and the novel Rhizobiales bacterium (KR911840-KR911843) are found in the bacteriomes of both sexes and in the ovaries; 2) *S*. *muelleri* is also found in the testes and the “filter chamber + conical segment” of the alimentary canal; and 3) *Rickettsia* sp. (KR911839) is found in the salivary glands, midgut, Malpighian tubules, and testes (Table 4).

**Table 4:** Distribution of four dominant bacteria in different tissues of *P*. *kaempferi*.

	Bacteriomes				Ovaries				Salivary glands				Filter + Conical segment				Midgut				Hindgut				Malpighian tubules				Testes			
	S	H	R	SP	S	H	R	SP	S	H	R	SP	S	H	R	SP	S	H	R	SP	S	H	R	SP	S	H	R	SP	S	H	R	SP
**PF1**	+	+	-	-	+	+	-	-	-	-	+	-	+	-	+	-	-	-	+	-	-	-	-	-	-	-	+	-	/	/	/	/
**PF2**	+	+	-	-	+	+	-	-	-	-	+	-	+	-	-	-	-	-	+	-	-	-	-	-	-	-	+	-	/	/	/	/
**PF3**	+	+	-	-	+	+	-	-	-	-	+	-	+	-	+	-	-	-	+	-	-	-	-	-	-	-	+	-	/	/	/	/
**PM1**	+	+	-	-	/	/	/	/	-	-	+	-	+	-	-	-	-	-	+	-	-	-	-	-	-	-	+	-	+	-	+	-
**PM2**	+	+	-	-	/	/	/	/	-	-	+	-	+	-	-	-	-	-	+	-	-	-	-	-	-	-	+	-	+	-	+	-
**PM3**	+	+	-	-	/	/	/	/	-	-	+	-	+	-	-	-	-	-	+	-	-	-	-	-	-	-	+	-	+	-	+	-

Abbreviation: S, *Sulcia muelleri*; H, the novel Rhizobiales bacterium; R, *Rickettsia* symbiont of *Nephotettix cincticeps*; SP, *Spiroplasma* sp.; PF, female *P*. *kaempferi*; PM, male *P*. *kaempferi*; MF, female *M*. *mongolica*; MM, male *M*. *mongolica*. The numbers 1, 2 and 3 represented the number of individual cicadas; +, presence; -, absence

Among the bacteria harbored in tissues of *M*. *mongolica*, *S*. *muelleri* is found in the bacteriomes of both sexes, ovaries, “filter chamber + conical segment” of the alimentary canal, and testes; *Rickettsia* sp. (KR911839) is found in the midgut and salivary glands; *Spiroplasma* sp. (KR911845 and KR911846) is found in the bacteriomes, midgut, and hindgut (Table 5).

**Table 5:** Distribution of four dominant bacteria in different tissues of *M*. *mongolica*.

	Bacteriomes				Ovaries				Salivary glands				Filter + Conical segment				Midgut				Hindgut				Malpighian tubules				Testes			
	S	H	R	SP	S	H	R	SP	S	H	R	SP	S	H	R	SP	S	H	R	SP	S	H	R	SP	S	H	R	SP	S	H	R	SP
**MF1**	+	-	-	+	+	-	-	-	-	-	+	-	+	-	-	-	-	-	+	+	-	-	-	+	-	-	-	-	/	/	/	/
**MF2**	+	-	-	+	+	-	-	-	-	-	-	-	+	-	-	-	-	-	+	+	-	-	-	+	-	-	-	-	/	/	/	/
**MF3**	+	-	-	+	+	-	-	-	-	-	-	-	+	-	-	-	-	-	+	+	-	-	-	+	-	-	-	-	/	/	/	/
**MM1**	+	-	-	+	/	/	/	/	-	-	+	-	+	-	-	-	-	-	+	+	-	-	-	+	-	-	-	-	+	-	-	-
**MM2**	+	-	-	+	/	/	/	/	-	-	-	-	+	-	-	-	-	-	+	+	-	-	-	+	-	-	-	-	+	-	-	-
**MM3**	+	-	-	+	/	/	/	/	-	-	-	-	+	-	-	-	-	-	+	+	-	-	-	+	-	-	-	-	+	-	-	-

Abbreviation: S, *Sulcia muelleri*; H, the novel Rhizobiales bacterium; R, *Rickettsia* symbiont of *Nephotettix cincticeps*; SP, *Spiroplasma* sp.; PF, female *P*. *kaempferi*; PM, male *P*. *kaempferi*; MF, female *M*. *mongolica*; MM, male *M*. *mongolica*. The numbers 1, 2 and 3 represented the number of individual cicada; +, presence; -, absence

### Illumina sequencing data of bacteriomes and reproductive organs

**Fig 4:**
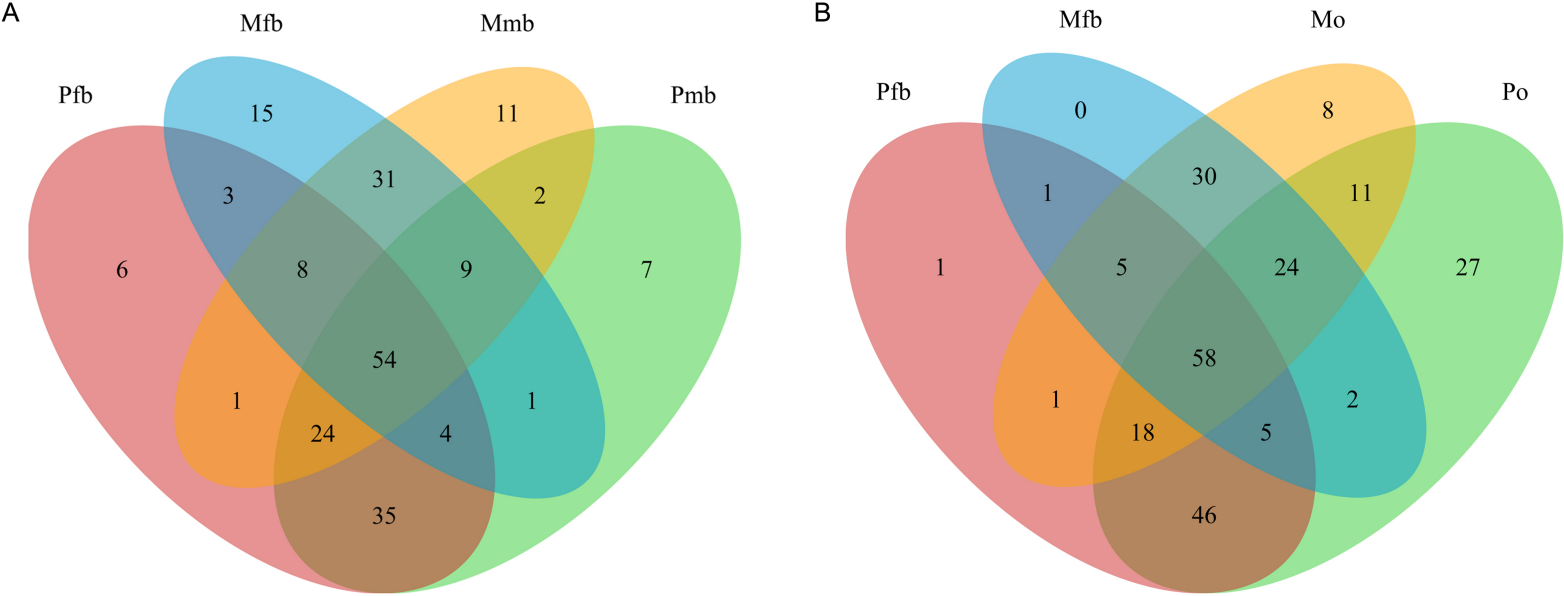
Venn diagrams showing OTUs shared among different tissues of *P*. *kaempferi* and *M*. *mongolica*. The numbers represent the number of unique OTUs owned by each sample and common OTUs shared by samples. A. OTUs of the bacteriomes of male and female *P*. *kaempferi* and *M*. *mongolica*. B. OTUs of the bacteriomes and ovaries of female *P*. *kaempferi* and *M*. *mongolica*. Abbreviations: Pfb, the bacteriomes of female *P*. *kaempferi*; Pmb, the bacteriomes of male *P*. *kaempferi*; Po, the ovaries of *P*. *kaempfer*; Mfb, the bacteriomes of female *M*. *mongolica*; Mmb, the bacteriomes of male *M*. *mongolica*; Mo, the ovaries of *M*. *mongolica*.

The number of high-quality sequences and bacterial OTUs obtained from the bacteriomes of females and males, ovaries and testes are shown in Table 6. The mean read length of the 16S rRNA variable V4 region of our samples is 274 bp. Venn diagrams show that 54 bacterial OTUs are shared among the bacteriomes of females and males of the two cicada species (Fig 4A). The bacteriomes of females share 117 and 102 bacterial OTUs with corresponding bacteriomes of conspecific males of the two cicada species, respectively (Fig 4A). The bacteriomes of females of *P*. *kaempferi* and *M*. *mongolica* share 127 and 117 bacterial OTUs with corresponding conspecific ovaries (Fig 4B).

**Table 6:** Sample information, sequence abundance, and bacterial diversity of bacteriomes and reproductive organs of *P*. *kaempferi* and *M*. *mongolica*.

Cicada species	Tissues	samples	No. high quality reads	No. OTUs	Richness indices		Diversity indices	
					Chao 1	ACE	Simpson	Shannon
***P*. *kaempferi***	Female’s bacteriomes	Pfb 1	51,485	92	147.5	157.6881	0.856569	3.192923
		Pfb 2	53,121	73	102.5455	113.0062	0.873599	3.32091
		Pfb 3	54,495	110	117.5	119.1729	0.855916	3.277058
	Male’s bacteriomes	Pmb 1	43,567	110	124.5263	129.3729	0.815574	3.055795
		Pmb 2	55,929	75	107.5	128.506	0.799739	2.88899
		Pmb 3	54,684	92	137.1111	130.2236	0.788628	2.994976
	Ovaries	Po 1	57,297	140	148.0526	151.0711	0.873436	3.555705
		Po 2	41,054	150	167.1429	159.1242	0.900093	4.189643
		Po 3	34,930	125	137.0476	141.5689	0.856592	3.323685
	Testes	Pt 1	31,747	154	165.6667	163.7039	0.524556	2.722502
		Pt 2	13,847	157	160.1111	159.7743	0.936305	5.080345
		Pt 3	36,555	115	144.3333	123.0031	0.57624	2.409039
***M*. *mongolica***	Female’s bacteriomes	Mfb 1	63,785	94	189.1429	143.2077	0.07151	0.371214
		Mfb 2	47,603	84	111.0833	109.4708	0.054173	0.29595
		Mfb 3	47,097	54	112	113.6921	0.027139	0.146286
	Male’s bacteriomes	Mmb 1	58,000	113	134.2308	134.0915	0.251171	0.99472
		Mmb 2	48,469	58	94.90909	109.4057	0.179517	0.592633
		Mmb 3	42,441	64	97.83333	109.13	0.227967	0.745928
	Ovaries	Mo 1	49,768	114	135	130.882	0.146561	0.732699
		Mo 2	42,236	103	120	115.5269	0.250143	1.084584
		Mo 3	52,681	97	116	124.9382	0.787206	3.350045
	Testes	Mt 1	43,068	125	146.5652	150.9444	0.139344	0.632187
		Mt 2	38,874	79	107.1111	103.8147	0.236368	0.844845
		Mt 3	38,484	119	150	152.7778	0.570017	2.150375

**Fig 5:**
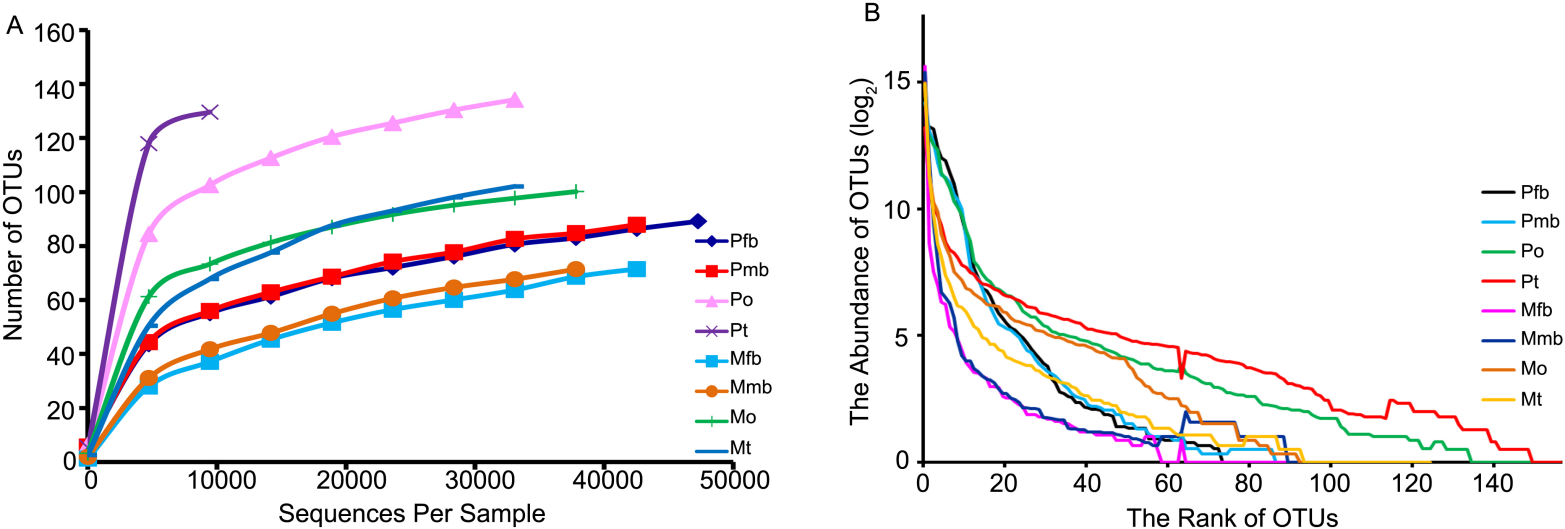
Analysis of bacterial OTUs in the bacteriomes and reproductive organs of *P*. *kaempferi* and *M*. *mongolica*. (A) Rarefaction curve. (B) rank-abundance curve. Abbreviations: Pfb, the bacteriomes of female *P*. *kaempferi*; Pmb, the bacteriomes of male *P*. *kaempferi*; Po, the ovaries of *P*. *kaempfer*; Pt, the testes of *P*. *kaempferi*; Mfb, the bacteriomes of female *M*. *mongolica*; Mmb, the bacteriomes of male *M*. *mongolica*; Mo, the ovaries of *M*. *mongolica*; Mt, the testes of *M*. *mongolica*.

Rarefaction curves of bacterial OTUs of *P*. *kaempferi* and *M*. *mongolica* show low slopes at high-sampling depth (Fig 5A), indicating that the sequencing method reliably represents the actual bacterial communities. The rank-abundance curves (Fig 5B) indicate that only the ovaries and testes of *P*. *kaempferi* contain a relatively high abundance of bacteria.

### Bacterial composition of bacteriomes and reproductive organs of *P*. *kaempferi* based on the Illumina sequencing data

**Fig 6:**
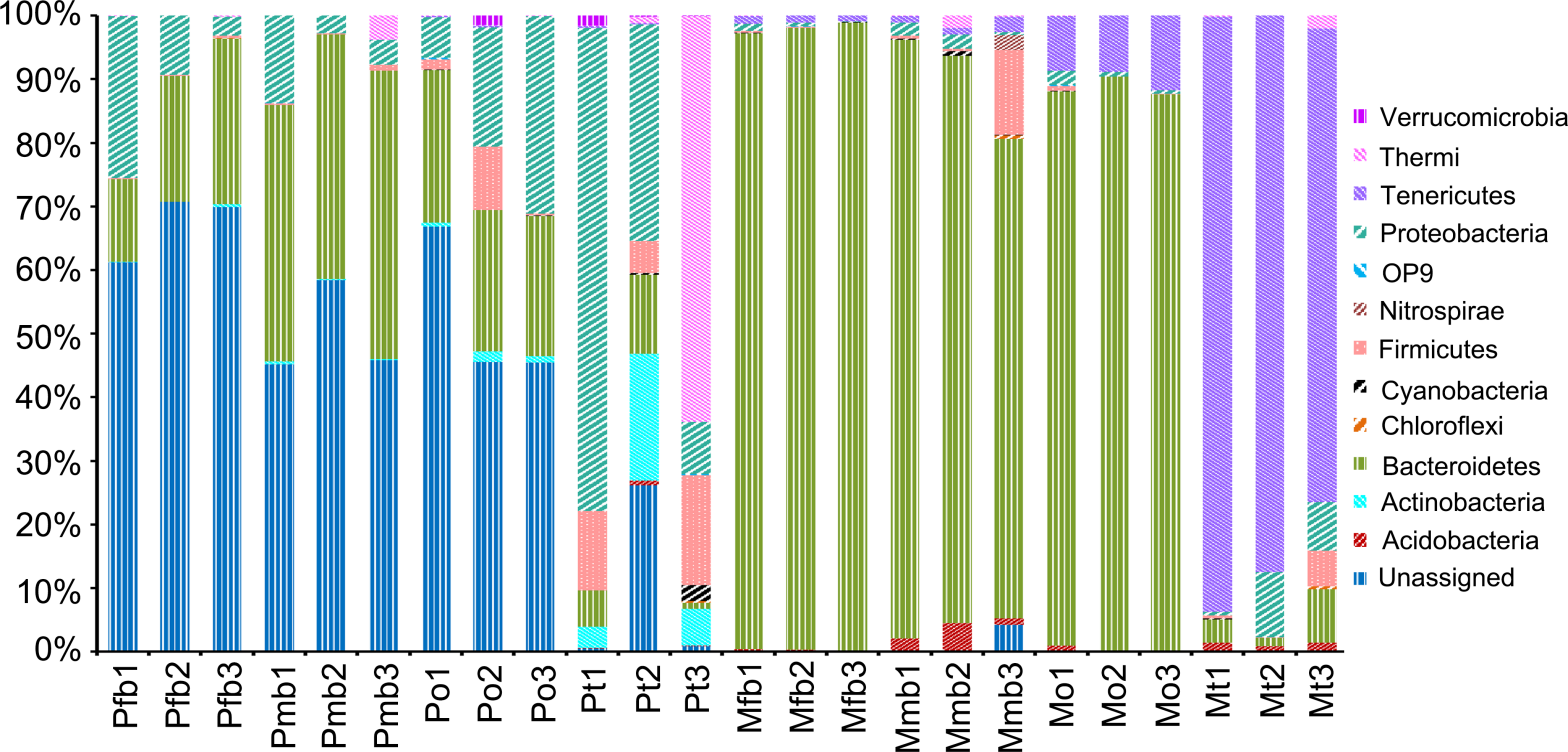
Bacterial composition of the bacteriomes and reproductive organs in *P*. *kaempferi* and *M*. *mongolica* at the phylum level. Abbreviations: Pfb, the bacteriomes of female *P*. *kaempferi*; Pmb, the bacteriomes of male *P*. *kaempferi*; Po, the ovaries of *P*. *kaempfer*; Pt, the testes of *P*. *kaempferi*; Mfb, the bacteriomes of female *M*. *mongolica*; Mmb, the bacteriomes of male *M*. *mongolica*; Mo, the ovaries of *M*. *mongolica*; Mt, the testes of *M*. *mongolica*. The numbers 1, 2 and 3 represented the three biological replicates for each sample.

The identified sequences of *P*. *kaempferi* are distributed across 12 assigned bacterial phyla, two archaebacteria phyla, and a large number of unassigned phyla (Fig 6). Bacterial community composition varies among different tissues of this cicada species. Bacteroidetes is dominant in the bacteriomes of both sexes (30.47 ± 12.82%) and ovaries (22.74 ± 1.04%), and is more abundant in the bacteriomes of males (41.36 ± 3.51%) than in those of females (19.59 ± 6.58%). Proteobacteria is the subdominant phylum in the bacteriomes of both sexes (9.69 ± 8.86%) and ovaries (18.71 ± 12.34%), and it is the most dominant bacterial phylum in the testes (39.43 ± 34.17%). The abundances of Actinobacteria and Firmicutes are greater in the testes (9.70 ± 8.91% and 11.58 ± 6.15% for Actinobacteria and Firmicutes, respectively) than in the bacteriomes of both sexes (0.22 ± 0.18% and 0.37 ± 0.27% for Actinobacteria and Firmicutes, respectively) and ovaries (1.10 ± 0.49% and 3.95 ± 5.21% for Actinobacteria and Firmicutes, respectively). Thermi is the dominant phylum in one testis sample (Pt3, 63.79%) but has a relatively low abundance in other samples. The remaining phyla (Actinobacteria, Firmicute, Acidobacteria, Chloroflexi, Cyanobacteria, Nitrospirae, OP9, Tenericutes and Verrucomicrobia) all have a low abundance.

**Fig 7:**
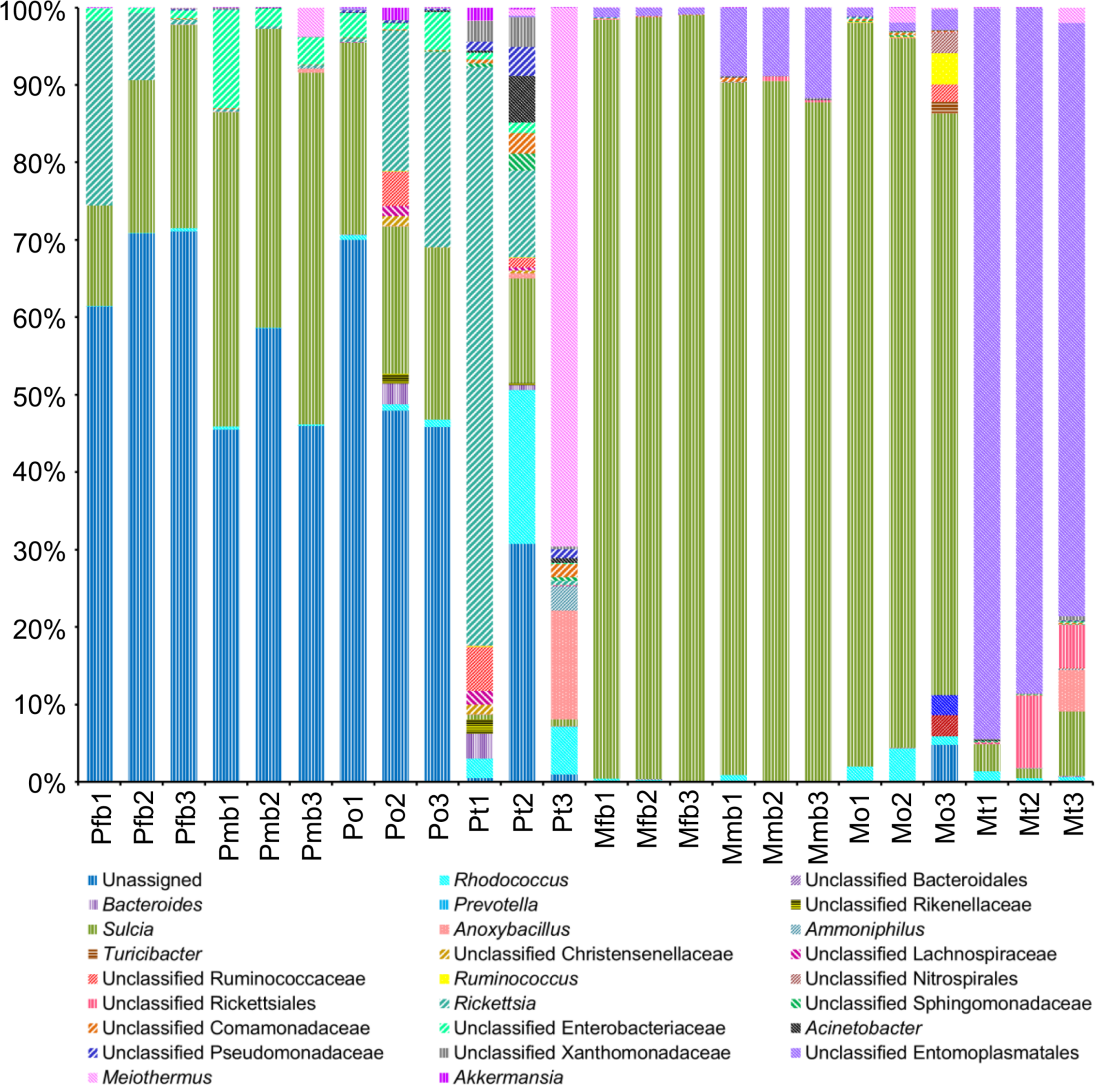
Bacterial composition of the bacteriomes and reproductive organs of *P*. *kaempferi* and *M*. *mongolica* at the genus/species level (sequence frequencies >1%). Abbreviations: Pfb, the bacteriomes of female *P*. *kaempferi*; Pmb, the bacteriomes of male *P*. *kaempferi*; Po, the ovaries of *P*. *kaempfer*; Pt, the testes of *P*. *kaempferi*; Mfb, the bacteriomes of female *M*. *mongolica*; Mmb, the bacteriomes of male *M*. *mongolica*; Mo, the ovaries of *M*. *mongolica*; Mt, the testes of *M*. *mongolica*. The numbers 1, 2 and 3 represented the three biological replicates for each sample.

At the genus/species level (Fig 7), *S*. *muelleri* is dominant in the bacteriomes of both males (19.47 ± 4.63%) and females (41.35 ± 0.73%) and ovaries (21.23 ± 1.72%); but it is relatively rare in the testes (4.32 ± 2.64%). *Rickettsia* is subdominant in the ovaries (14.19 ± 12.53%) and the bacteriomes of females (11.01 ± 11.77%), and is dominant in one testis sample (Pt1, 68.74%). Moreover, *Rhodococcus* (19.66%), *S*. *muelleri* (11.23%) and *Rickettsia* (9.27%) are dominant in Pt2; *Meiothermus* (63.42%) is dominant in Pt3; *Rhodococcus*, *Rickettsia* and *Meiothermus* are present at a low level in the bacteriomes of both sexes and ovaries. Minor genera, including *Bacteroides*, *Lactococcus*, *Phascolarctobacterium*, *Acinetobacter* and *Akkermansia*, are present in low percentages.

Of particular note, the sequences of unclassified bacteria reach 56.56 ± 11.15% in the bacteriomes of both sexes and ovaries (Fig 7). We further identified the unclassified OTUs by blasting their sequences with the GenBank database, and found that 90% of the unclassified OTUs are similar to *H*. *cicadicola* with an identity value of ~80%, which is the same as the sequence alignment results of the novel Rhizobiales bacterium (KR911840-KR911843) obtained by the RFLP. The sequences of *Rickettsia* and *Meiothermus* obtained by high throughput sequencing also show a high similarity respectively with the *Rickettsia* sp. (KR911839) and *Meiothermus cerbereus* (KR911844) obtained by the RFLP, both with identity values of 96–99%.

### Bacterial composition of bacteriomes and reproductive organs of *M*. *mongolica* based on the Illumina sequencing data

The bacteria identified in *M*. *mongolica* are classified into 11 bacterial phyla, two archaebacteria phyla, and a small proportion of unassigned phyla (Fig 6). Bacteroidetes is dominant in the bacteriomes of females (97.78 ± 0.99%) and males (88.28 ± 1.76%), and ovaries (86.21 ± 9.72%). Tenericutes is dominant in the testes (85.18 ± 9.75%), and is subdominant in the bacteriomes of females (9.70 ± 1.73%) and males (1.07 ± 0.15%), and ovaries (1.52 ± 0.74%). Proteobacteria is subdominant in the testes (6.13 ± 4.96%), but occurs in a low percentage in the bacteriomes of females (0.62 ± 0.54%) and males (1.21 ± 0.91%), and ovaries (1.56 ± 0.95%). Firmicutes contributes 13.26% and 5.66% of sequences for Mo3 and Mt3, respectively, but is rare (less than 1%) in other samples. Minor phyla (Actinobacteria, Chloroflexi, Cyanobacteria, Firmicutes, Nitrospirae, OP9, Thermi and Verrucomicrobia) all exhibit a relatively low frequency (less than 1%) in all the samples.

At the genus/species level (Fig 7), *S*. *muelleri* is dominant in the bacteriomes of females (97.70 ± 0.74%) and males (67.49 ± 13.80%), and ovaries (83.18 ± 7.64%). It is subdominant in the testes (41.99 ± 3.44%) where an unclassified bacterium of Entomoplasmatales (85.18 ± 5.39%) is dominant. The sequences of the unclassified Entomoplasmatales are closely similar to 16S rRNA sequences of the genus *Spiroplasma* in NCBI, with identity values of 95–99%, which possess identity values of 85–99% with the sequences of *Spiroplasma* sp. (KR911845 and KR911846) obtained by the RFLP. The sequences of *Meiothermus* and *Rhodococcus* obtained by the high throughput sequencing all show a high similarity with *Meiothermus cerbereus* (KR911849) and *Rhodococcus* sp. (KR911850) obtained by the RFLP, respectively, all with identity values of 96–99%.

### Bacterial diversity of bacteriomes and reproductive organs based on the Illumina data

A bioinformatic analysis was applied to evaluate the Alpha-diversity (Shannon, Simpson, Chao 1, and ACE indices) and Beta-diversity of bacterial communities of all samples of *P*. *kaempferi* and *M*. *mongolica*.

**Fig 8:**
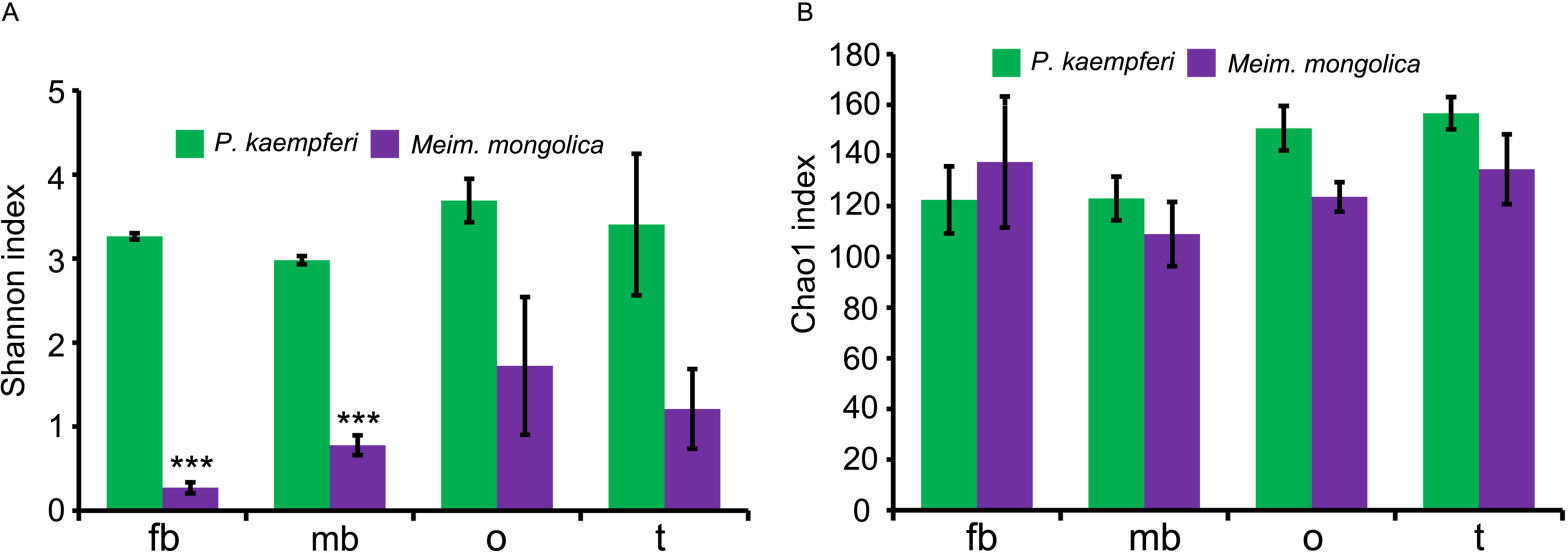
Diversity indices of the bacterial communities of the bacteriomes and reproductive organs of *P*. *kaempferi* and *M*. *mongolica*. (A) Differences of Shannon index. (B) Differences of Chao 1 index. Abbreviations: fb, the bacteriomes of females; mb, the bacteriomes of males; o, the ovaries; t, the testes. Differences of diversity indices were analyzed by employing ANOVA analysis and Fisher’s Least Significant Difference (LSD) post hoc test (* *P* <0.05, ** *P* <0.01, *** *P* <0.001).

According to the Shannon index, the bacterial diversity shows no difference among the investigated tissues of *P*. *kaempferi* (Table 6 and Fig 8A). The bacterial diversity of ovaries (1.72 ± 1.42) and testes (1.21 ± 0.82) is higher than that of bacteriomes of females (0.27 ± 0.11) and males (0.78 ± 0.20) in *M*. *mongolica*, but without significance (Table 6 and Fig 8A). The bacterial diversity of bacteriomes of both sexes, ovaries and testes of *P*. *kaempferi* is higher than that of corresponding tissues of *M*. *mongolica* (Table 6 and Fig 8A), but the bacterial diversity of bacteriomes of *P*. *kaempferi* is significantly higher than that of *M*. *mongolica* (*P* <0.05) (Fig 8A).

Based on the Chao 1 index, the bacterial richness of ovaries and testes of *P*. *kaempferi* is higher than that of bacteriomes of the same gender, and the bacterial richness of testes is significantly higher than that of bacteriomes of both sexes (*P* <0.05) (Table 6 and Fig 8B). The bacterial richness of bacteriomes of *M*. *mongolica* is the lowest, while, no significant difference is found among different tissues (Table 6 and Fig 8B). The bacterial richness of corresponding tissues of the two cicada species shows that the bacteriomes of males, ovaries and testes of *P*. *kaempferi* are all higher than that of corresponding tissues of *M*. *mongolica* (123.05 ± 14.86 vs 108.99 ± 21.91, 150.75 ± 15.23 vs 123.67 ± 10.02, and 156.70 ± 11.07 vs 134.56 ± 23.83, respectively), apart from the bacteriomes of females where it is just the opposite (122.52 ± 22.89 vs 137.41 ± 44.81) (Table 6 and Fig 8B), but this difference is again not significant (Fig 8B).

**Fig 9:**
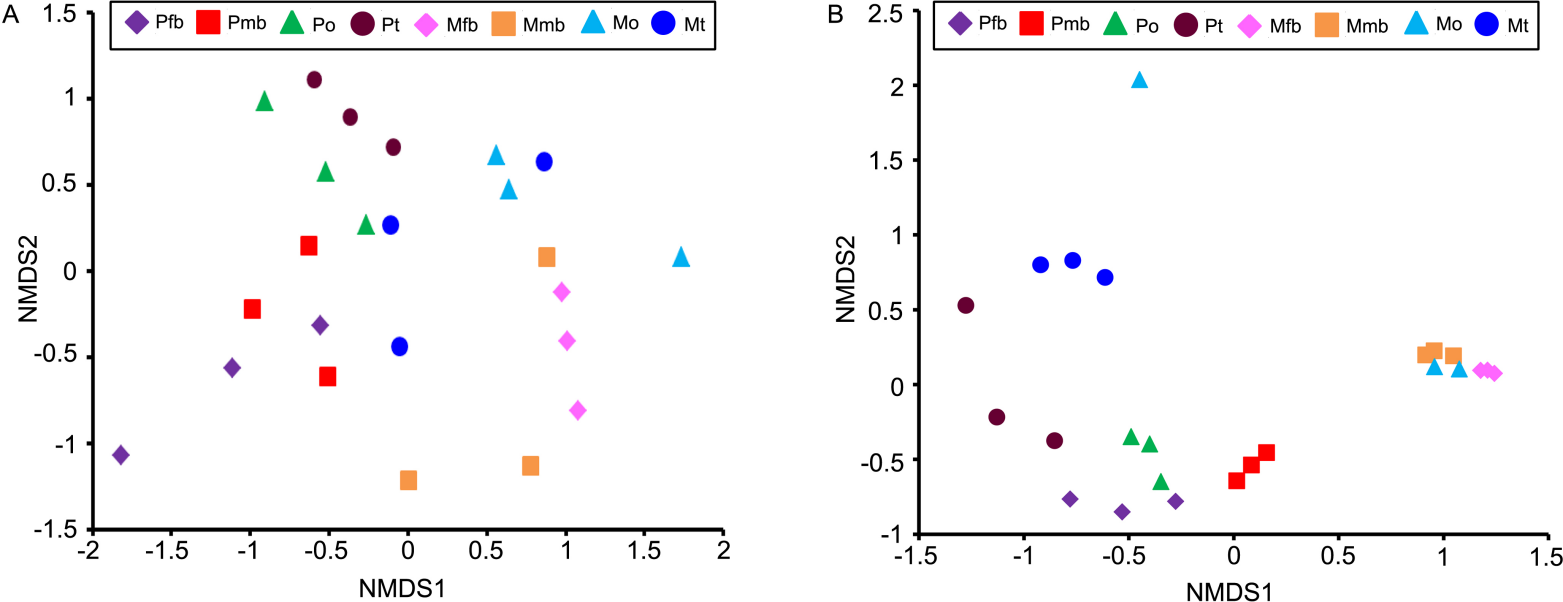
Non-metric multidimensional scaling (NMDS) ordination plots of bacterial community structures across different individual samples of *P*. *kaempferi* and *M*. *mongolica*. (A) unweighted NMDS analysis (B) weighted NMDS analysis. Abbreviations: Pfb, the bacteriomes of female *P*. *kaempferi*; Pmb, the bacteriomes of male *P*. *kaempferi*; Po, the ovaries of *P*. *kaempfer*; Pt, the testes of *P*. *kaempferi*; Mfb, the bacteriomes of female *M*. *mongolica*; Mmb, the bacteriomes of male *M*. *mongolica*; Mo, the ovaries of *M*. *mongolica*; Mt, the testes of *M*. *mongolica*.

Unweighted nonmetric multidimensional scaling (NMDS) (stress = 0.12) (Fig 9A) did not reveal distinct clusters in either of the cicada species. In contrast, the weighted analysis (stress = 0.08) (Fig 9B) shows that the bacteriomes and ovaries of *P*. *kaempferi* form a cluster at the bottom-left, with conspecific testes loosely close by while the bacteriomes and ovaries of *M*. *mongolica* form a tight cluster at the middle-right. Conspecific testes and an ovary sample are extremely dispersed from the cluster. This shows that bacterial communities in the bacteriomes and ovaries of *P*. *kaempferi* are distinctly different from those of *M*. *mongolica*, and that bacterial communities of the bacteriomes have no correlation with the sex of these related cicada species.

### Nucleotide sequence accession numbers

The 16S rRNA gene clone sequences detected using RFLP are deposited in GenBank (NCBI) under the accession numbers KR911839–KR911844 and KR911845–KR911850, respectively. Sequence data obtained using Illumina high-throughput sequencing are deposited to the Sequence Read Archive (NCBI) under the accession Bioproject number PRJNA315940.

## Discussion

### Comparison of 16S rRNA RFLP and high-throughput sequencing

In this study, we detected more bacterial species in the bacteriomes of female *P*. *kaempferi* and *M*. *mongolica* using the high-throughput sequencing rather than using RFLP. This shows that the former technology is more powerful in detecting bacteria. Nevertheless, it has some shortcomings. For instance, most of the sequences with a similarity of <97% cannot be annotated. Also, the bacterial community abundance estimated directly using read frequencies might be inaccurate, as sequences from some bacterial species may be more likely to be amplified than those of other species [33]. Despite being less powerful in detecting bacteria, the RFLP approach can detect dominant bacteria and provide their complete 16S rRNA sequences, which may facilitate subsequent experiments, e.g., diagnostic PCR and FISH.

### The primary symbionts associated with cicadas

The endosymbiont *S*. *muelleri* is dominant in the bacteriomes and ovaries of both cicada species. As has been reported, *S*. *muelleri* is an a-symbiont of Auchenorrhyncha [34], which has co-evolved with the lineage for more than 260 million years [6], and has been retained in most descendant lineages but lost in some [35]. This bacterium has been observed in the bacteriomes of different leafhoppers [36,37], planthoppers [38], and cicadas including *Diceroprocta semicincta* (Davis), *Magicicada septendecim* (Linnaeus) and *Tettigetta mariae* (Quartau & Boulard) [5,39]. Genomic analyses on *S*. *muelleri* in the glassy-winged sharpshooter *Homalodisca vitripennis* (Germar) and the periodical cicada *Magicicada septendecim* revealed that it can provide eight of the 10 essential amino acids (arginine, phenylalanine, tryptophan, lysine, threonine, isoleucine, leucine, and valine) to its insect hosts [2]. The distribution patterns of *S*. *muelleri* in our current study confirm the transovarial transmission of this primary endosymbiont between insect generations. To date, the cellular mechanism for vertical transmission of obligate bacteria in bacteriomes has been studied thoroughly for *Buchnera* in the pea aphid *Acyrthosiphon pisum* (Harris) [4], but more studies are required to clarify the vertical transmission mechanism of related endosymbionts in cicadas. For the first time, *S*. *muelleri* was found not to be confined to the bacteriomes and reproductive organs, but was shown to occur in the “filter chamber + conical segment” of the alimentary canal of both cicada species. A previous study reporting morphological and ultrastructural observations on the alimentary canal of *P*. *kaempferi* did not reveal the presence of typical symbiont cells in the “filter chamber + conical segment” [40], which was possibly due to the bacterium not appearing in the images shown in that study. Thus, further study is needed to confirm the distribution and transmission mode of *S*. *muelleri* in the alimentary canal of cicadas and its effect on insects’ physiological processes.

### The novel Rhizobiales bacterium

*Sulcia muelleri* typically co-occurs with another bacterium that varies by insect groups, e.g., with *H*. *cicadicola* in cicadas [2], *Candidatus* Baumannia cicadellinicola in sharpshooters [12], and *Candidatus* Zinderia insetticola in spittlebugs [29]. McCutcheon and Moran [29] found that *H*. *cicadicola* could produce methionine and histidine for cicadas. Genomic analyses of the tiny *H*. *cicadicola* genome found that it has split into two new cytologically distinct but metabolically interdependent parts in some but not all species in the cicada genus *Tettigades* [41]. In our present study, partial clones and unclassified OTUs harbored in the bacteriomes and ovaries of *P*. *kaempferi* are affiliated with Rhizobiales and mostly similar to *H*. *cicadicola* (NR_074753.1) with an identity value of ~80% (Fig 3 and S1 Fig). Given that the genome of *H*. *cicadicola* was previously shown to exhibit a rapid rate of sequence evolution [42], it is possible that the novel Rhizobiales species is a variant with beneficial trophic functions similar to that of *H*. *cicadicola* reported in some cicadas of the genera *Magicicada*, *Diceroprocta* and *Tettigades* [30,41]. Our failure to detect *H*. *cicadicola* in *M*. *mongolica* is possibly because this symbiont has been replaced by another bacterium, a yeast or gut microbiota which compensates for the loss of *H*. *cicadicola* [35]. This hypothesis merits further research. The distribution of the novel Rhizobiales species in other cicadas and its identification also require further investigation to help establish the definition of the bacterial genus *Candidatus* Hodgkinia and to provide more information on sequence divergence of the primary endosymbionts of cicadas.

### The secondary symbionts associated with cicadas

A large number of secondary symbionts were also detected in our study. For example, *Rickettsia* sp. (KR911839), previously documented from the green leafhopper *Nephotettix cincticeps*, is found in the bacteriomes of both sexes, salivary glands, midgut, ovaries and testes of *P*. *kaempferi*, and is also found in the salivary glands and midgut of *M*. *mongolica*. This bacterium has also been identified from the bacteriomes of the pea aphid *Acyrthosiphon pisum* [43], and the reproductive organs, digestive and salivary glands of the whitefly *Bemisia tabaci* (Gennadius) [18]. The infection of *Rickettsia* in *Bemisia tabaci* was hypothesized to contribute to producing the gelling saliva required for stylet penetration into plant tissue, and to play a possible role in food digestion [18]. The infection of *Rickettsia* sp. (KR911839) in the salivary glands and midgut of *P*. *kaempferi* and *M*. *mongolica* may help facilitate xylem-sap intake and digestion. Future studies are required to clarify the exact functions of *Rickettsia* sp. in Cicadidae.

*Spiroplasma* sp. (KR911845 and KR911846) was also detected in *M*. *mongolica*. This bacterium has been reported to associate with a wide range of insects, e.g., some species of *Spiroplasma* were pathogenic for honeybees, fruit flies (*Drosophila*), mosquitos, and moths [44], and some were mutualists in leafhoppers, fruit flies (*Drosophila*) and aphids [43,45,46]. *Spiroplasma* was usually in a low concentration in insect guts, suggesting that they do not replicate in the gut or invade insect cells as do to other nonpathogenic gut bacteria, and that they may have nutritional or other symbiotic roles [47,48]. The function of *Spiroplasma* sp. harbored in the midgut and hindgut of *M*. *mongolica* needs investigation.

The genus *Rhodococcus* was detected from both cicada species in our study. Members of this genus have been found in blood-sucking bugs of the genus *Triatoma*, the parasitic fly *Wohlfahrtia magnifica* (Schiner) [49,50], and the leafhopper *Homalodisca vitripennis* [51]. *Rhodococcus rhodnii* is an endosymbiont of the bug *Rhodnius prolixus* (Stål) and may supply the bug with B vitamins. Bugs of the same species lacking this endosymbiont die prematurely during nymphal development [52]. Thus, *Rhodococcus *in cicadas may supply the hosts with some nutrients, but this requires confirmation.

*Bacillus* sp. (KR911847) and *Anoxybacillus*, both affiliated with Bacillaceae, were also identified in our study. *Bacillus* sp. and *Anoxybacillus* were reported to supplement digestive enzymes in degrading xylan, cellulose and phenolic components in lignin, which improve access to nutrients by their insect hosts [53,54]. Whether the related bacteria harbored in cicadas have similar trophic functions remains unknown.

*Meiothermus cerbereus* (KR911844 and KR911849) was detected at a relatively low concentration in the bacteriomes of females of both *P*. *kaempferi* and *M*. *mongolica* using RFLP, but it was not detected in any tissues of *M*. *mongolica* using high-throughput sequencing. This is probably due to the extremely low concentration of this bacterium in some samples. *Meiothermus cerbereus* is a thermophilic bacterium that has been reported as a dominant bacterium mostly in warm fresh-water environments [55]. *Meiothermus* produces restriction enzymes which are more tolerant of extreme conditions of temperature and pH [56]. *Meiothermus cerbereus* may have no influence on cicadas, or it may increase the fitness and thermostability of cicadas, in particular under stressful environmental conditions in summer. However, the exact function of this bacterium in cicada species needs investigation. The mode of infection of cicadas by this bacterium also merits further study.

The remaining bacterial taxa such as Enterobacteriaceae, *Bacteroides*, *Acinetobacter* and *Sphingobium* are only found at low frequencies in some of the investigated samples. Their effects are also unknown.

### The composition and diversity of bacterial communities in bacteriomes and reproductive organs

The bacterial community composition, Alpha-diversity and Beta-diversity analyses in the present study imply that, within a cicada species, the bacterial communities of the testes are significantly different from those of bacteriomes and ovaries. Moreover, the bacterial communities of corresponding tissues between the two cicada species are significantly different. This discrepancy could be due to certain factors. First, some bacterial species may only reside in a specific host. Second, the interactions of bacteria within the host can dramatically affect the dynamics of bacterial population and, therefore, impact the evolution of the host-symbiont interaction and modify parameters such as host resistance and co-evolution with the host [57]. Third, cicadas may live in different ecosystems/niches and feed on different host plants; e.g., *P*. *kaempferi* mainly feeds on xylem sap of pines, cypresses and poplars, while *M*. *mongolica* mainly feeds on poplars [58,59]. And fourth, variations of bacterial communities, particularly among secondary endosymbionts, may occur among individual samples. Thus, the two cicada species contain significantly diverse bacterial communities.

## Conclusion

In conclusion, despite the types of technologies used in detecting bacterial communities, our study mainly provides qualitative results of bacterial community composition and diversity in the bacteriomes and reproductive organs of *P*. *kaempferi* and *M*. *mongolica*. We also clarify the distribution of four dominant bacterial species in the digestive and excretory systems of these two cicada species. Further studies should focus on the following unresolved issues by using other technologies, e.g., FISH, quantitative real-time PCR, RNA-Seq and genomic sequencing: first, the impact of bacterial communities on their cicada hosts at the individual, population and species levels; second, the function of each representative bacterial species, particularly the primary and secondary endosymbionts in cicadas (e.g., *S*. *muelleri* and the novel Rhizobiales bacterium which shows ~80% similarity to *H*. *cicadicola*); third, the co-evolution between bacterial communities and their cicada hosts.

## Materials and methods

### Ethics statement

No specific permits were required for this study. This study did not involve endangered or protected species, and the cicadas *Platypleura kaempferi* and *Meimuna mongolica* used in the present study was not included in the “List of Protected Animals in China”.

### Dissection of insect samples and DNA extraction

Adults of *P*. *kaempferi* used for RFLP were collected at the Huoditang Experimental Forest Station of the Northwest A & F University, Ningshan County, Shaanxi Province, China, in July of 2014. Adults of *M*. *mongolica* used for RFLP were captured at Yangling, Shaanxi Province, China, in August of 2014. Then in 2015, adults of both sexes of these two cicada species were separately captured during the adult mergence period at the same location as in 2014 for high throughput sequencing. About 20–30 individual cicadas for each species were captured by using a light trap at each time. Specimens were kept in centrifuge tubes stored at 4°C, and transported to the laboratory for vivisection as soon as possible. Female and male insect samples were surface sterilized with 75% ethanol for 3 min, and rinsed in sterile water several times, then dissected under sterile conditions under a Stereoscopic Zoom Microscope (Motic SMZ168, Xiamen, China). The bacteriomes, ovaries and testes were carefully pulled apart without rupturing with sterile forceps, respectively. Between dissecting different organs, forceps were flame-sterilized to protect against cross-contamination between organs. Dissected organs were then washed with sterile water several times, and individually placed in 1.5 ml collection tubes and stored at −80°C for further analysis. Three replicate samples were taken for each tissue.

Each sample of *P*. *kaempferi* and *M*. *mongolica* individuals was treated with lysozyme and incubated for 24 h, and total genomic DNA of all samples were respectively extracted with the DNeasy Blood and Tissue Kit (Tiangen Inc.), according to the manufacturer’s instructions. DNA extracts were stored at −20°C until further analysis.

### RFLP analysis

The DNA samples from bacteriomes of females of *P*. *kaempferi* and *M*. *mongolica* were amplified by PCR using the universal primers 27F (5′-AGAGTTTGATCCTGGCTCAG-3′) and 1492R (5′-GGTTACCTTGTTACGACTT-3′) [60]. PCR was performed in a 25 μl reaction mixture, consisting of 1 μl Template DNA, 2.5 μl 10× PCR Buffer, 1.5 μl 25 mM MgCl_2_, 2 μl 2.5 mM dNTP Mixture, and 0.25 μl 5 U/μl Taq DNA polymerase, 1 μl 10 mM of each primer, and 15.75 μl dd H_2_O. PCR thermal profile was 94°C for 2 min, followed by 30 cycles, with each cycle consisting of 94°C for 30 s, 55°C for 45 s, and 72°C for 1 min. After cycling, a final extension was carried out at 72°C for 10 min.

The amplified fragments were purified with a PCR purification kit (Tiangen Inc.) and inserted into pMD^®^19-T Vector (Tiangen Inc.). The ligated mixture was transformed into *Escherichia coli* DH5α (Tiangen Inc.) competent cells. For each sample, about 200 white clones were randomly selected and used as a template for PCR with M13 forward and reverse primers to check the positive clones. The PCR products of positive clones were digested respectively with *Afa* I and *Hha* I restriction endonucleases (Takara Bio. Inc.) in 37°C for 4 h, then the restriction fragment length polymorphism (RFLP) patterns were separated by 1.5% agarose gel electrophoresis. The restriction profiles were then compared and grouped and one to three representative clones for each unique RFLP profile were sequenced at Sangon Biotech Co., Ltd (Shanghai, China).

All representative clones were sequenced in both forward and reverse directions, and the sequences were manually trimmed to remove the sequences of plasmid and primers, and assembled using the SeqMan program (DNAStar, Inc.). All clones with >97% identity were defined as the same operational taxonomic unit (OTU), as 97% sequence identity is the criterion used to identify bacteria at the species level. Then, represented sequences were blasted in GenBank and the Ribosomal Database Project (http://rdp.cme.msu.edu/) to find their closest relatives.

Diversity indices were calculated using the software SPADE (http://chao.stat.nthu.edu) to evaluate the diversity of bacteria in the two bacteriome-clone libraries of females. Rarefaction curves were conducted using the software Analytic Rarefaction version 1.3 (http://strata.uga.edu) to assess the adequacy of bacteria in the bacteriomes of female *P*. *kaempferi* and *M*. *mongolica*, and the default parameters were used. Two Maximum Likelihood (ML) trees were constructed with the Kimura 2-parameter model and 2000 bootstrap replicates in MEGA 5.0 [61] after all sequences were checked and best matched sequences aligned in Clustal X [62].

### Diagnostic PCR

In order to reveal the distribution of dominant bacteria associated with bacteriomes of females detected by RFLP in other tissues (i.e., bacteriomes in males, ovaries, salivary glands, foreguts, midguts, hindguts, Malpighian tubules and testes), three female and three male individuals of both cicada species were processed for diagnostic PCR. PCR primers targeting the 16S rRNA gene sequences of different dominant bacteria were used: 10_CFB_FF (5’-AGAGTTTGATCATGGCTCAGGATG-3’) and 1515_R (5’-GTACGGCTACCTTGTTACGACTTAG-3’) for *S*. *muelleri* [6]; HG-F1 (5’-GAACYGTAAAMCTCTTTTGYCRR-3’) and HG-R2 (5’-GAGCTAGCTTTCGCTTGGAAG-3’) for the novel Rhizobiales bacterium; NcRic_16S/f1 (5’-TGACGGTACCTGACCAAGA-3’) and NcRic_16S/r1 (5’-AAGGGATACATCTCTGCTT-3’) for *Rickettsia* sp. [36]; SP-F1 (5’-GTAAGYAWAGGAAATGWRYTTAT-3’) and SP-R2 (5’-CRGTTGCRATCTYGTAAGAGG-3’) for *Spiroplasma* sp. HG-F1 and HG-R2, SP-F1 and SP-R2 were designed by primer 5.0. PCR was performed in a 25 μl reaction mixture, consisting of 1 μl Template DNA, 2.5 μl 10× PCR Buffer, 1.5 μl 25 mM MgCl_2_, 2 μl 2.5 mM dNTP Mixture, and 0.25 μl 5 U/μl Taq DNA polymerase, 1 μl 10 mM of each primer, and 15.75 μl dd H_2_O. PCR thermal profile was 95°C for 5 min, followed by 35 cycles, with each cycle consisting of 94°C for 30 s, 55°C for 30 s, and 72°C for 1 min. After cycling, a final extension was carried out at 72°C for 7 min. The annealing temperature and time should be modulated for different bacterial species.

### Illumina high-throughput sequencing preparation

Genomic DNA of the bacteriomes of both sexes and reproductive organ samples of *P*. *kaempferi* and *M*. *mongolica* individuals were amplified by primers 520F (5’-GCACCTAAYTGGGYDTAAAGNG-3’) and 802R (5’-TACNVGGGTATCTAATCC-3’), targeting their 16S rRNA hypervariable V4 region. A 25 μl reaction system was prepared for PCR mixtures and the PCR products were visualized by using 2% agarose gel electrophoresis and purified using a PCR purification kit (Tiangen Inc.). The purified PCR products were quantified and pooled, and sent for sequencing on the Illumina MiSeq platform (Personal Biotechnology Co., Ltd, Shanghai, China), according to the protocols described by Caporaso [63].

### Sequence data analyses

After sequencing, sequences were trimmed and assembled by Flash (version 1.2.7, http://ccb.jhu.edu/software/FLASH/) [64], and the reads which could not be assembled were discarded. Chimeras were identified and removed using Uchime (Mothur) (version 1.31.2, http://www.mothur.org/) [65]. The cleaned Fastq data were aligned into operational taxonomic units (OTUs) by uclust (QIIME) based on a similarity of 97% [66]. Then, taxonomy was assigned using the BLAST algorithm against the Greengenes database (Release 13.8, http://greengenes.secondgenome.com/) [67]. The sequences of unclassified OTUs were blasted against the GenBank database of NCBI. Furthermore, the sequences obtained by the RFLP were blasted against those obtained by the high throughput sequencing. The rarefaction curves and Alpha diversity indices (Ace, Chao 1, Shannon and Simpson indices) were plotted using the mothur package (QIIME), and we performed ANOVA and Fisher’s Least Significant Difference (LSD) post hoc test on Chao 1 and Shannon indices respectively by using the SPSS 18.0 software. Beta diversity was also used to evaluate the degree of similarity of bacterial communities associated with different tissues (bacteriomes, ovaries, testes) and cicada species (*P*. *kaempferi* and *M*. *mongolica*) by using QIIME to calculate Unifrac distances (http://bmf2.colorado.edu) [68,69]. Finally, a nonmetric multidimensional scaling (NMDS) analysis was performed.

## Supporting information

S1 Fig The ML phylogenetic tree based on 16S rRNA gene sequences of the novel Rhizobiales bacterium obtained from the bacteriomes of female *P*. *kaempferi*, including selected sequences of Rhizobiales of insects in the GeneBank. This tree was generated using the Maximum Likelihood with 2,000 bootstrap replicates and Kimura 2-parameter model in MEGA5.0 software. The four representative clones of the novel Rhizobiales bacterium are presented with dark spots followed by GenBank accession numbers. The scale bar represents 0.05 substitutions per nucleotide site.
(TIF)
