# Supplementary figures and images for: Bacterial diversity of bacteriomes and organs of reproductive, digestive and excretory systems in two cicada species (Hemiptera: Cicadidae)
Source: PLoS One. 2017 Apr 24;12(4):e0175903. doi: 10.1371/journal.pone.0175903 (PMC5402938; doi:10.1371/journal.pone.0175903)

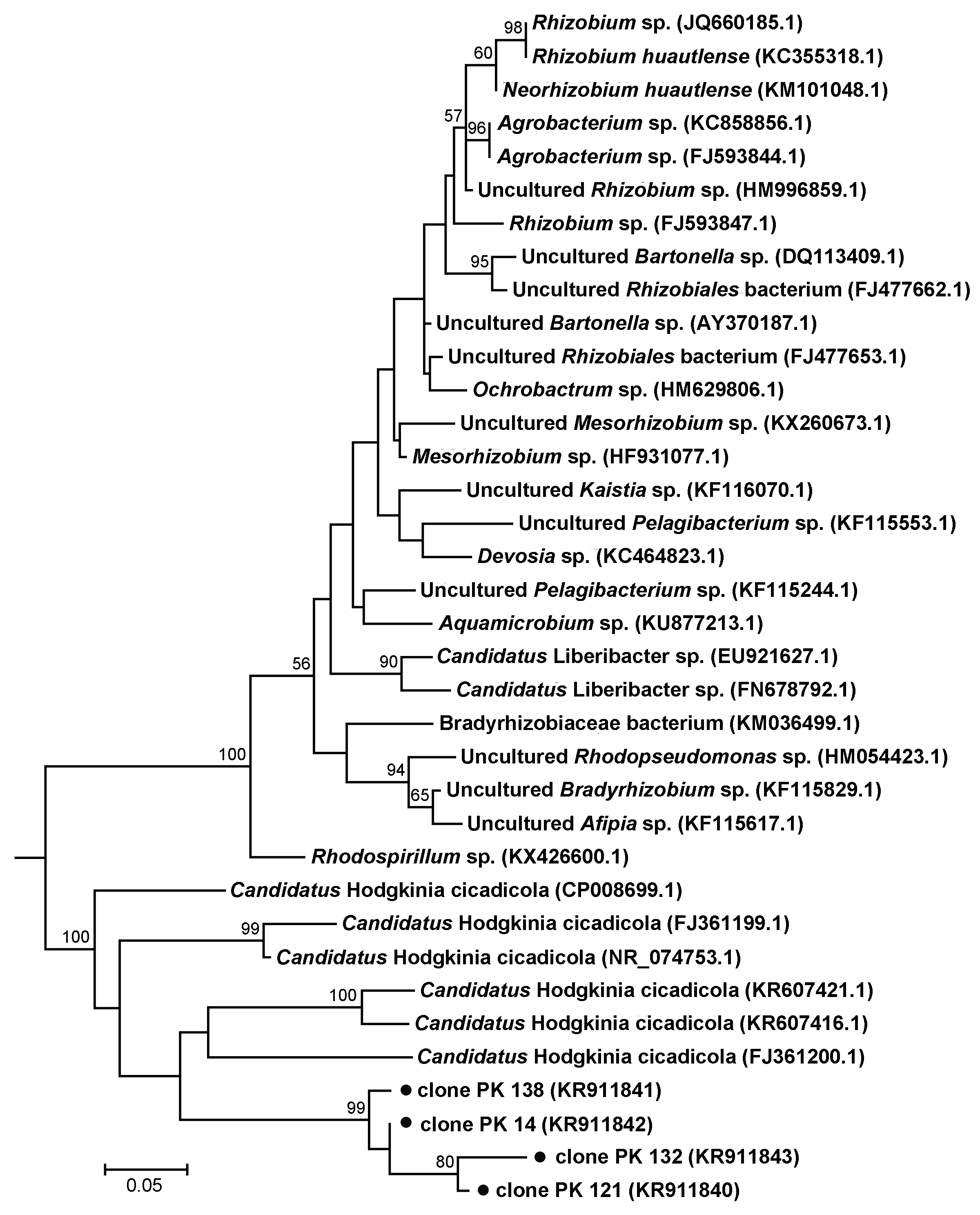

Supplement: S1 Fig — This tree was generated using the Maximum Likelihood with 2,000 bootstrap replicates and Kimura 2-parameter model in MEGA5.0 software. The four representative clones of the novel Rhizobiales bacterium are presented with dark spots followed by GenBank accession numbers. The scale bar represents 0.05 substitutions per nucleotide site. (TIF) [file pone.0175903.s001.tif]
